# Special Issue: ECG Monitoring System

**DOI:** 10.3390/s21020651

**Published:** 2021-01-19

**Authors:** Florent Baty

**Affiliations:** Lung Center, Cantonal Hospital St. Gallen, 9000 St. Gallen, Switzerland; florent.baty@kssg.ch

This editorial of the Special Issue “ECG Monitoring System” provides a short overview of the 13 contributed articles published in this issue.

In the first paper, Bae and collaborators [1] propose an innovative methodology for the detection of R-points in ECG measurements. The method was then successfully applied for the efficient analysis and detection of major arrhythmias.

In the second paper, Lackner and colleagues [2] provide detailed guidance and recommendations on the interpretation of heart rate variability (HRV) analysis from ECG data monitored over long periods. The main findings reveal that time-domain variables are mostly adequate to describe individual’s HRV from long-term ECG monitoring. On the other hand, the selection and use of frequency-domain variables require more careful considerations.

In the third paper, Holmes and colleagues [3] explore HRV measurements from photoplethysmography (PPG) via smartphone app and compare them with ultrashort-term ECG in adults before and after resistance exercise. They conclude that smartphone PPG is comparable to ECG for measuring HRV at rest, but is prone to larger errors when measured during exercise.

In another manuscript, Satti et al. [4] introduce a rigid parylene-coated microneedle electrode array coupled with a portable ECG circuit. The newly designed wearable wireless ECG monitoring device reduces the noise due to movement artifacts and, as such, proves to be particularly useful for ECG recording during dynamic behaviors.

Tang and colleagues [5] discuss motion artifacts inherent in wearable capacitive ECG acquisition and propose a new capacitive electrode for controllable humidification. They could verify the validity and performance of the proposed design, which can effectively suppress motion artifacts and maintain stability of the signal quality at various levels of ambient humidity.

Klum and collaborators [6] employed a multimodal patch stethoscope to estimate Einthoven ECG Lead I and II from a single 55 mm ECG lead. They conclude that the estimation of ECG, together with the pre-ejection period and left ventricular ejection time as well as respiratory parameters, is feasible using a wearable, multimodal acquisition device.

Another manuscript from Wang et al. [7] explores the premise of automatic detection of arrhythmia based on the multiresolution representation of ECG signals.

Baty and colleagues [8] developed a novel wearable ECG belt which provides high accuracy for the assessment of sleep apnea severity.

Wang and collaborators [9] propose an innovative low-power high-data-transmission multilead ECG acquisition sensor system. The vest-type acquisition system is suitable for continuous operation over a long period of time, and is useful for clinical diagnosis.

Nie and co-workers [10] discuss the original concept of continuous heart rate monitoring of livestock. Their study indicates that systems based on PPG sensors are feasible for implementation in livestock, with continuous and accurate heart rate monitoring.

The review of Serhani and colleagues [11] presents a comprehensive and systematic overview of ECG monitoring systems. This thorough review of the literature highlights the challenges and current trends associated with this new technology.

Ohmuta et al. [12] developed a new ECG recognition technique using wavelet transformation, which is useful for precise automated QT-interval measurements.

Finally, Al-Karadi and collaborators [13] discuss the challenges associated with the identification of U wave abnormalities from ECG signals. They specifically applied a multibeat averaging algorithm to efficiently measure and characterize U waves from healthy subjects in pre- and post-exercise recordings.
